# A Randomized Clinical Trial of a Fractional Low Dose of BNT162b2 Booster in Adults Following AZD1222

**DOI:** 10.3390/vaccines10060914

**Published:** 2022-06-08

**Authors:** Rapisa Nantanee, Watsamon Jantarabenjakul, Peera Jaru-Ampornpan, Pimpayao Sodsai, Orawan Himananto, Jitthiwa Athipunjapong, Jiratchaya Sophonphan, Sira Nanthapisal, Nattiya Hirankarn, Thanyawee Puthanakit

**Affiliations:** 1 Center of Excellence in Pediatric Infectious Diseases and Vaccines, Department of Pediatrics, Faculty of Medicine, Chulalongkorn University, Bangkok 10330, Thailand; rapisa.n@chula.ac.th (R.N.); watsamon.j@chula.ac.th (W.J.); jitthiwa.a@chula.ac.th (J.A.); 2 Pediatric Allergy and Clinical Immunology Research Unit, Division of Allergy and Immunology, Department of Pediatrics, Faculty of Medicine, Chulalongkorn University, Bangkok 10330, Thailand; 3 Thai Red Cross Emerging Infectious Diseases Clinical Center, King Chulalongkorn Memorial Hospital, Bangkok 10330, Thailand; 4 Virology and Cell Technology Research Team, National Center for Genetic Engineering and Biotechnology (BIOTEC), Pathum Thani 12120, Thailand; peera.jar@biotec.or.th; 5 Center of Excellence in Immunology and Immune-Mediated Diseases, Department of Microbiology, Faculty of Medicine, Chulalongkorn University, Bangkok 10330, Thailand; pimpayao.s@chula.ac.th; 6 Monoclonal Antibody Production and Application Research Team, National Center for Genetic Engineering and Biotechnology (BIOTEC), Pathum Thani 12120, Thailand; orawanh@biotec.or.th; 7 The HIV Netherlands Australia Thailand Research Collaboration (HIV-NAT), The Thai Red Cross AIDS Research Centre, Bangkok 10330, Thailand; jiratchaya.w@hivnat.org; 8 Department of Pediatrics, Faculty of Medicine, Thammasat University, Pathum Thani 12120, Thailand; nsira@tu.ac.th; 9 Department of Microbiology, Faculty of Medicine, Chulalongkorn University, Bangkok 10330, Thailand; nattiya.h@chula.ac.th

**Keywords:** SARS-CoV-2 vaccine, booster dose, neutralizing antibody titer, anti-SARS-CoV-2 IgG, BNT162b2 vaccine, ChAdOx1 nCoV-19 vaccine, AZD1222, SARS-CoV-2 omicron variant, heterologous prime-boost COVID-19 vaccination

## Abstract

In the era of globally predominant omicron strains, a COVID-19 booster vaccine is needed. Our study aimed to evaluate the immunogenicity of a half-dose BNT162b2 booster after AZD1222 in healthy adults. A randomized trial of volunteers aged 18–69 years who received two-dose AZD1222 was conducted. The participants were randomized to receive the BNT162b2 vaccine intramuscularly—half (15 µg) vs. standard dose (30 µg). The immunogenicity was evaluated by a surrogate virus neutralization test (sVNT) against omicron variants and anti-spike-receptor-binding-domain IgG (anti-S-RBD IgG). From November–December 2021, 100 adults with a median age of 59.3 years (IQR 33.4–65.5) were enrolled. A booster dose was given at median of 98 days (IQR 92–128) after AZD1222. At day 14, the geometric means (GMs) of anti-S-RBD IgG in half- vs. standard-dose group were 2329.8 vs. 2574.7 BAU/mL, with a geometric mean ratio (GMR) of 0.90 (0.77–1.06). The GMs of sVNT against the omicron variant in the half- and standard-dose groups were 74.4% inhibition (95% CI 68.8–80.5) and 67.3% inhibition (57.9–78.1), respectively, with GMR of 0.95 (0.69–1.30). At day 90, the sVNT indicated 22.3% inhibition (95% CI 14.9–33.4) and 20.4% inhibition (13.1–32.0), respectively, with GMR of 1.09 (0.60–1.98). The fractional low-dose BNT162b2 mRNA booster vaccine provided non-inferior immunogenicity responses. During a shortage of vaccine supply, a fractional low dose should be considered for a booster vaccination program.

## 1. Introduction

Since the COVID-19 pandemic, several variants of concern of SARS-CoV-2 have emerged. The delta variant (B.1.617.2) was the predominant circulating variant from June to December 2021 [1]; the omicron variant (B.1.1.529) has replaced it as a dominant global variant since January 2022 [2]. This variant has been shown to more rapidly spread compared with previous variants, and transmission has also occurred among vaccinated or previously SARS-CoV-2-infected individuals [2]. The omicron variant contains 15 mutations in the receptor-binding domain (RBD) in the spike protein [3], which might contribute to immune evasion, as shown by the reduced neutralizing activities from post-vaccination or previous-infection sera [4,5], and lower effectiveness of previous infection [6]. COVID-19 vaccine effectiveness wanes over time, as shown by the increased rate of infection, and symptomatic and severe disease [7]. Considering both the changes in the virus and waning of antibodies leading to a decline in vaccine effectiveness, a booster dose is needed, especially in at-risk groups, e.g., elderly and immunocompromised persons [8]. Booster doses were shown to reduce COVID-19-associated emergency department visits and hospitalizations during both periods of delta and omicron predominance, with vaccine effectiveness for hospitalizations of 93% for three-dose mRNA vaccination vs. 82% for two doses [9].

Heterologous prime-boost vaccination, a vaccination with a combination of different vaccine platforms, have demonstrated robust immunogenicity and good safety profiles for both primary-series [10] and boosting regimens [11]. The adenoviral vectored vaccine ChAdOx1 nCoV-19 (Oxford–AstraZeneca; AZD1222) and the mRNA vaccine BNT162b2 (Pfizer–BioNTech) have been shown to be effective [12,13] and rolled out worldwide, including in Thailand. Based on data from the COV-BOOST study from the UK, a standard-dose BNT162b2 booster after two-dose AZD1222 priming showed higher antibody and cellular response than a homologous booster with AZD1222, with SARS-CoV-2 anti-spike IgG geometric mean ratios (GMRs) of 24.48 (95% CI 19.50–30.79) and 3.25 (2.52–4.20) compared with controls, respectively [11].

A fractional low dose of booster vaccine has been approved for mRNA-1273 (Spikevax, Moderna, Cambridge, MA, USA) [14]. Half-dose mRNA-1273 (50 µg) booster was demonstrated to boost neutralizing antibody titers higher than those after the completion of the primary series at 1 month, with GMR of 1.7 (95% CI 1.5–1.9) for the wild-type and 2.1 (95% CI 1.8–2.4) for the delta variant [15]. According to the COV-BOOST study, half-dose BNT162b2 booster showed comparable immunogenicity to the standard dose in AZD1222-primed individuals, with anti-spike IgG GMR of 16.80 (95% CI 12.97–21.76) and 24.48 (19.50–30.79) compared with controls, respectively [11], and anti-receptor binding domain (anti-RBD) IgG GMR of 7.04 (4.69–8.84) and 8.49 (5.71–10.44) compared with the post-primary series [16]. A low-dose BNT162b2 booster after AZD1222 also showed lower adverse events, e.g., fatigue, fever, and local reaction, than the standard-dose booster [16]. Based on the correlation between neutralizing antibody titers and vaccine efficacy against symptomatic infection [17], a model using data from dose-finding trials suggested that half or one-fourth doses of mRNA-1273 have 90 to 95% efficacy, similar to a standard dose [18]. One-third and two-third doses of BNT162b2 also showed predicted efficacy of 70 to 85%, compared with 95% for a standard dose [18]. Using a fractional dosing strategy might ease the limited vaccine supply, thus speeding up vaccine coverage and reducing mortality, even if it yields lower efficacy [18].

This study aimed to compare the immunogenicity of half- and standard-dose BNT162b2 vaccine boosters in healthy adults post-AZD1222 primary immunization.

## 2. Materials and Methods

### 2.1. Study Design and Participants

This study was conducted at the Faculty of Medicine, Chulalongkorn University, Bangkok, Thailand. This was a double-blinded, randomized, controlled trial in healthy adults aged 18–69 years old, who received 2-dose AZD1222 for more than 60 days, with an interval of at least 8 weeks apart. Any participants receiving blood products within 3 months, any vaccines (within 2 weeks for inactivated vaccines or 4 weeks for live vaccines), or with previous SARS-CoV-2 infection, by history or positive anti-nucleocapsid antibody, were excluded. All participants signed written informed consent prior to study enrollment.

This study was registered in the Thai Clinical Trials Registry (thaiclinicaltrials.org, TCTR20211027002). The Institutional Review Board of the Faculty of Medicine, Chulalongkorn University (IRB No. 873/64) approved this study.

### 2.2. Study Procedures

The participants were stratified according to their age—adult, 18–59 years of age, and elderly, 60–69 years of age, then randomized correspondingly to include equal participants of both age groups in each arm due to potentially different antibody responses, as in the previous report [19]. The randomization process was performed using a block of four in a sealed envelope. The participants were vaccinated with intramuscular BNT162b2, 0.45 mL per vial (6 doses per vial), lot number FK6268; 0.15 mL (15 µg, half dose); or 0.3 mL (30 µg, standard dose). Unblinded nurses performed vaccination in a participant-blinded fashion. Therefore, the participants and blinded study team were not aware of the randomization arms. During the first week post-vaccination, the solicited local and systemic reactogenicity were logged by the participants using a diary. Unsolicited adverse events were also described by the study team at every visit.

Blood samples were obtained prior to booster vaccination (day 0), and 14 days and 90 days post-booster-vaccination. All samples were tested for spike receptor binding domain IgG (anti-S-RBD IgG) and a surrogate virus neutralization test (sVNT) was conducted. At baseline, sVNT against B.1.617.2 (delta variant) and the wild type was evaluated. With the rapid spread of B.1.1.529 (omicron variant) in Thailand and globally, sVNT against the omicron variant was assessed at day 14 and day 90, along with the delta variant at all time points and the wild type at day 14.

The enzyme-linked immunospot (ELISpot) assay was conducted in 20 random participants per group at day 0, day 14, and day 90 to evaluate the cell-mediated immune response.

### 2.3. Immunogenicity Outcomes

#### 2.3.1. Quantitative Spike Receptor Binding Domain IgG (Anti-S-RBD IgG) ELISA

The ELISA protocol was adapted from Amanat et al. (2020) [20]. Briefly, diluted serum samples were incubated in 96-well plates coated with purified recombinant Myc-His-tagged S-RBD residues 319–541 from SARS-CoV-2 (Wuhan-Hu-1). Then, ELISA was performed. The anti-S-RBD IgG level was reported in binding antibody units (BAU/mL) following the conversion of OD450 values with the standard curve using known units of the WHO international standard (NIBSC 20/136).

#### 2.3.2. Surrogate Virus Neutralization Test (sVNT)

A surrogate virus neutralization test was set up as previously described in Tan et al. (2020) [21]. Recombinant SRBD (residues 319–541) from the wild-type (Wuhan-Hu-1), delta (B.1.617.2), and omicron (B.1.1.529) strains and the ectodomain of human ACE2 were produced and purified from Human Embryonic Kidney (HEK) 293T cells transfected with expression plasmids carrying these genes. Serum samples (at 1:10 dilution)—SRBD mixture were incubated in 96-well plates coated with 0.1 µg/well recombinant human ACE2 ectodomain. Then, ELISA was performed. Pre-2019 human serum was used in the above procedure to give negative OD450. The % inhibition was calculated based on the ratio between negative OD450 and sample OD450. The % inhibition was calculated as follows:

$$\%\;\mathrm{inhibition}=100\times \left[1-\frac{\mathrm{sample}\;\mathrm{OD}450}{\mathrm{negative}\;\mathrm{OD}450}\right]$$

#### 2.3.3. Enzyme-Linked Immunospot (ELISpot) Assay

An ELISpot assay by a Human IFN-γ ELISpotPro^TM^ kit (Mabtech, Stockholm, Sweden) was used for assessing the T cell responses. Freshly isolated peripheral blood mononuclear cells (PBMCs) with 250,000 cells per well were activated with 2 µg/mL of overlapping peptide pool from 100 peptides of SARS-CoV-2 spike (S)-defined peptides (Mabtech, Stockholm, Sweden) for 20 h. A negative control and positive control, anti-CD3, were also included. The spots were quantified with an ImmunoSpot analyzer. To evaluate positive S peptide-specific responses, spot counts of negative control wells were subtracted from S peptide-stimulated wells, and these spot counts were reported as spot forming units (SFUs) per million PBMCs.

### 2.4. Reactogenicity

The solicited reactogenicity was recorded during the first week post-booster vaccination by self-recording in a diary. The solicited local reactogenicity included pain at the injection site, swelling, and erythema. The solicited systemic reactogenicity included fever, headache, fatigue, myalgia, arthralgia, vomiting, and diarrhea. Unsolicited adverse events were also described by the study team at every visit.

Adverse events were graded according to the Guidance for Industry Toxicity Grading Scale for Healthy Adult and Adolescent Volunteers Enrolled in Preventive Vaccine Clinical Trials, 2007 [22]: grade 0 for no symptoms; grade 1 for mild symptoms with no interference with activity, vomiting 1–2 times/day, or diarrhea 2–3 times/day; grade 2 for moderate symptoms with some interference with activity, vomiting more than 2 times/day, or diarrhea 4–5 times/day; grade 3 for severe symptoms, including incapacitation, vomiting/diarrhea requiring outpatient intravenous hydration, or diarrhea 6 or more times/day; and grade 4 for potentially life-threatening symptoms requiring emergency hospital visit or hospitalization. The grading of fever was grade 1 for 38.0–38.4 °C, grade 2 for 38.5–38.9 °C, grade 3 for 39–40 °C, and grade 4 for more than 40 °C. Feverish was defined as a feeling of fever but body temperature being less than 38.0 °C. Local reactogenicity, e.g., erythema and swelling, was graded by size: grade 1 for 2.5–5 cm, grade 2 for 5.1–10 cm, grade 3 for >10 cm, and grade 4 for necrosis.

### 2.5. Statistical Analysis

The sample size was calculated using a non-inferiority criterion for the geometric mean ratio (GMR) of sVNT against variants of concern, comparing the half dose with a standard dose. Assuming a GMR of 0.75 with 0.67 non-inferiority margin, 80% power, and a significance level of 0.05, the number of required participants was 22 per group. Considering a 10% loss-to-follow-up rate, the number of required participants was 25 per half-dose or standard-dose group in each age cohort (adult 18–59 years of age and elderly 60–69 years of age).

The demographic, laboratory data, and other continuous variables were presented as the median (interquartile range (IQR)), while the categorical variables were presented as the number with percentage. The Wilcoxon rank-sum test, chi-square, or Fisher’s exact test were calculated to determine the differences in the continuous and categorical variables between two groups, respectively. The primary endpoints, GMR of sVNT against variants of concern, and anti-S-RBD IgG, comparing the half- with the standard-dose groups at day 14 and 90 after booster, were compared in terms of non-inferiority. Non-inferiority was established if the lower bound of the 95% confidence interval (CI) of GMR was greater than 0.67 [23]. The geometric means (GMs) were calculated as the exponentiated means of the logarithmic transformation of the assay results. The two-sided 95% CIs were the antilogarithm of titers from the difference of two independent t-tests. The reactogenicity rates were compared using a chi-square test. All reported *p* values were two-sided. *p* values of <0.05 were considered to be statistically significant. Statistical analysis was performed using Stata version 15.1 (Stata Corp., College Station, TX, USA).

## 3. Results

### 3.1. Baseline Characteristics

From November to December 2021, 100 adults with a median age of 59.3 years (IQR 33.4–65.5) were enrolled. The median interval between AZD1222 was 79 days (IQR 70–84). The median time to booster doses was 98 days (IQR 92–128). Prior to the BNT162b2 booster, the GMs were 32.8% inhibition (95% CI 27.5–39.1) for sVNT against the delta variant, and 199.1 BAU/mL (170.1–233.0) for anti-S-RBD IgG. The baseline characteristics of the study participants are shown in Table 1. At day 90, 46 participants in both groups were tested for immunogenicity parameters due to 2 participants being lost to follow-up and 6 participants with breakthrough COVID-19 (3 in half-dose and 3 in full-dose, and 4 in 60–69-year-old group) with a range of 58–89 days post-booster during the omicron-predominant period, as shown in Supplementary Table S1. Four participants had mild symptoms of COVID-19 and two participants were asymptomatic.

### 3.2. Reactogenicity

**Figure 1:**
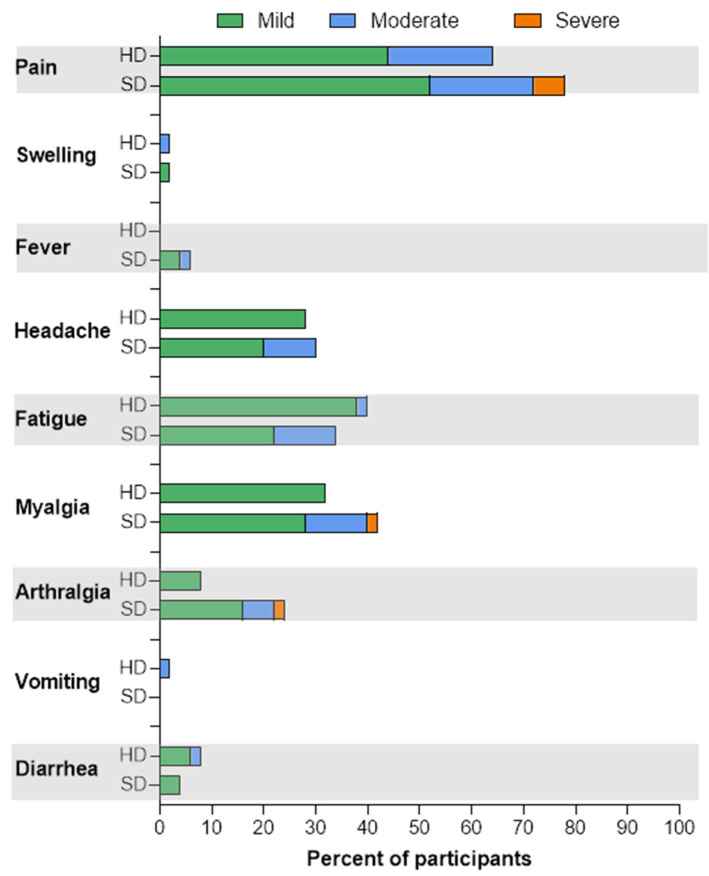
Local and systemic reactogenicities after the BNT162b2 booster in adults following 2 doses of AZD1222, according to half-dose and standard-dose groups. HD: Half dose; SD: Standard dose.

After BNT162b2 boosters, most participants had pain at the injection sites. Only the standard-dose recipients graded local pain as grade 3 (severe), as shown in Figure 1 and Supplementary Table S2. Fever also developed only in the full-dose arm. The full-dose recipients tended to have more moderate–severe systemic reactogenicities, e.g., myalgia, arthralgia, fatigue, and headache. Myalgia occurred more frequently in the standard-dose group (42% vs. 32% in the half-dose group, *p* 0.04).

### 3.3. Immunogenicity

#### 3.3.1. SARS-CoV-2-Neutralizing Antibody by Surrogate Virus Neutralization Test (sVNT)

**Figure 2:**
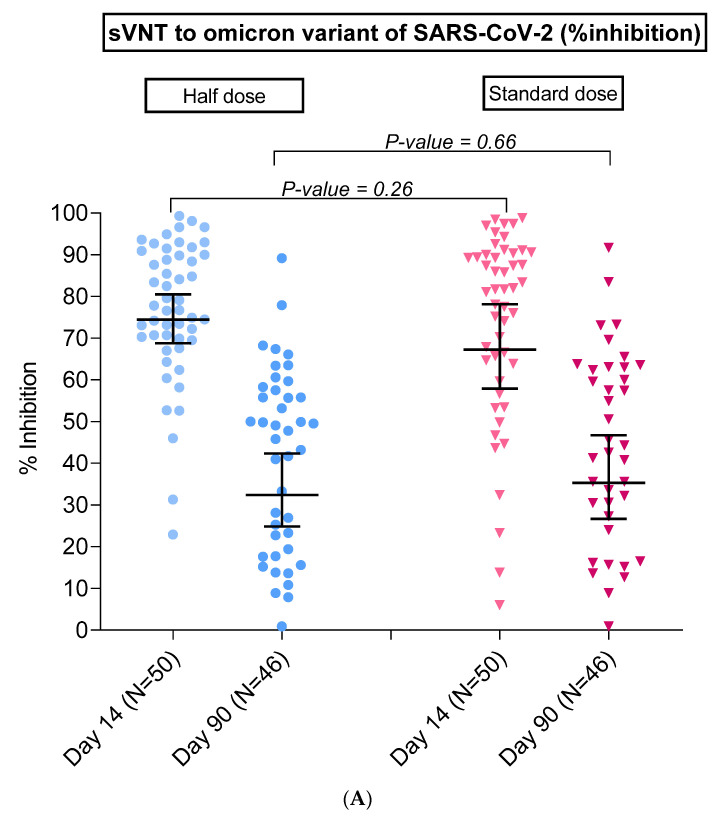

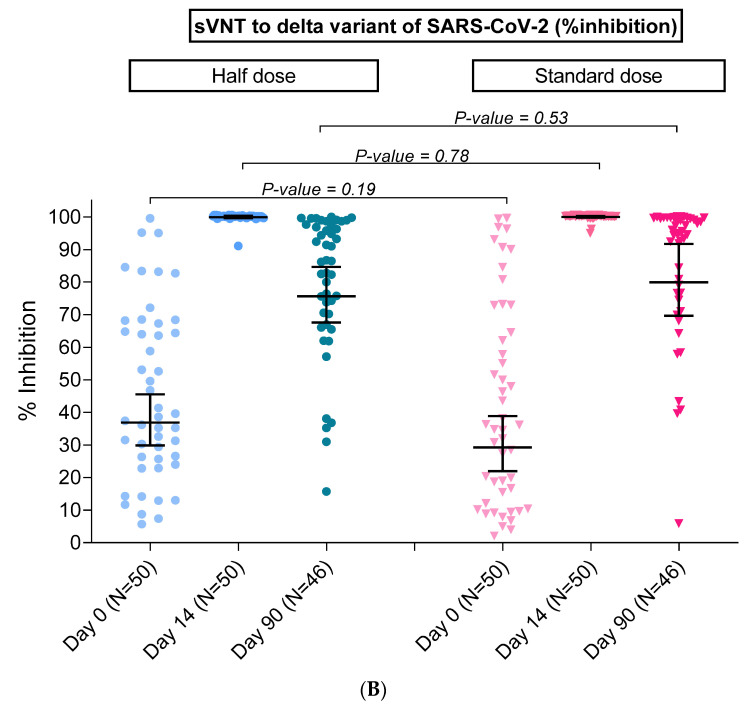
Geometric means (95% CI) of (**A**). sVNT for omicron variant of SARS-CoV-2 (% inhibition) at day 14 and day 90 after BNT162b2 booster doses, (**B**). sVNT for delta variant of SARS-CoV-2 (% inhibition) at day 0 (before BNT162b2 booster doses), day 14, and day 90 after booster doses, in adults post-2 doses of AZD1222, according to half-dose and standard-dose groups. sVNT: Surrogate virus neutralization test.

At day 14, the GMs of sVNT against the omicron variant were 74.4% inhibition (95% CI 68.8–80.5) post-half-dose BNT162b2 boosters, and 67.3% inhibition (57.9–78.1) post-standard-dose boosters, as shown in Table 2 and Figure 2A. The GMR of sVNT against the omicron variant was 1.11 (95% CI 0.93–1.30), showing the non-inferiority of the half-dose to the standard-dose booster. After 90 days, the neutralizing activity against the omicron variant declined, with GMs of 22.3% inhibition (95% CI 14.9–33.4) in the half-dose arm and 20.4% inhibition (13.1–32.0) in the standard-dose arm. The sVNT against the omicron variant in the half-dose vaccinees did not meet the pre-defined non-inferiority criteria to the standard dose, with GMR of 1.09 (95% CI 0.60–1.98).

The results of sVNT against the delta variant were higher than those against the omicron variant at the same time points. At day 14, both groups had sVNT against delta variant GMs of 100.0% inhibition, as shown in Table 2 and Figure 2B. At day 90, the sVNT against delta variant GMs slightly dropped, with 75.7% inhibition (95% CI 67.6–84.7) in the half dose and 79.9% inhibition (69.7–91.7) in the standard dose. GMRs of sVNT against the delta variant showed non-inferiority at both time points.

#### 3.3.2. SARS-CoV-2 Binding Antibody by Anti-S-RBD IgG

The anti-S-RBD IgG results, which were based on the RBD of the wild-type virus, showed a similar trend to sVNT. At day 14, anti-S-RBD IgGs were comparably boosted in the half- and standard-dose groups, with GMs of 2329.8 BAU/mL (95% CI 2109.3–2573.4) and 2574.7 BAU/mL (2262.5–2929.9), respectively, and GMR of 0.90 (0.77–1.06). At day 90, anti-S-RBD IgG waned and failed to meet predefined non-inferiority criteria, with GMR of 0.81 (95% CI 0.66–1.00). However, the quantitative assay of anti-S-RBD IgG was based on the RBD of the wild-type virus due to the lack of a universally recognized and standardized serum sample for anti-S-RBD IgG against other variants. Therefore, the levels of anti-S-RBD IgG might not well correlate with protection against the omicron variant.

In this study, participants were stratified and randomized according to their age—18–59 years and 60–69 years. Adults, aged 18–59 years, who received full-dose BNT162b2, had higher anti-S-RBD IgG than half-dose recipients, with GMs of 2837.3 BAU/mL (95% CI 2446.7–3290.3) and 2272.0 BAU/mL (1962.6–2630.2) at day 14 (*p* 0.03), respectively; and at day 90, 821.5 BAU/mL (666.4–1012.7) and 549.7 BAU/mL (446–677.4), *p* 0.01, respectively. However, no differential responses were seen in the other immunological outcomes and all immunological outcomes of the 60–69-year-old age group.

#### 3.3.3. Cell-Mediated Immune Response by ELISpot Assay

**Figure 3:**
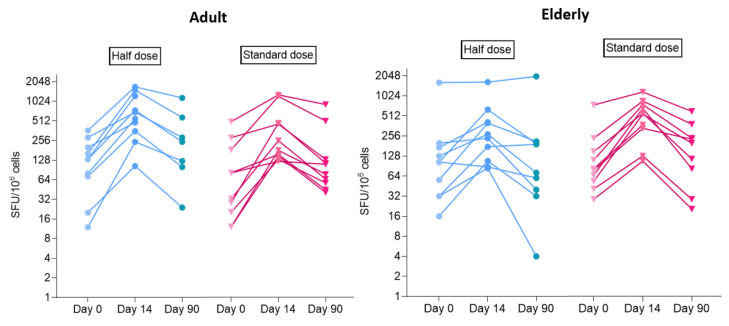
IFNγ ELISpot assay to detect S peptide-specific T-cell responses, presented as the median (IQR), at day 0 (before BNT162b2 booster doses), day 14, and day 90 after booster doses in adults post-2 doses of AZD1222, according to half-dose and standard-dose groups. SFU: spot forming unit; S: Spike of SARS-CoV-2. Adult: age 18–59 years; Elderly: age 60–69 years.

Prior to BNT162b2 boosters, the frequency of T cell responses to S peptide, as measured by the IFNγ ELISpot assay, was 80 SFU/10^6^ PBMCs (IQR 32–176) without statistically significant differences between the half- and standard-dose groups (half dose: median 116 SFU/10^6^ PBMCs, IQR 44–186; standard dose: median 78 SFU/10^6^ PBMCs, IQR 30–162; *p* 0.40), as shown in Figure 3. At 14 days after boosters, the T cell responses were significantly enhanced to 406 SFU/10^6^ PBMCs (IQR 206–718, *p* < 0.001) in the half-dose and 402 SFU/10^6^ PBMCs (148–678, *p* < 0.001) in the full-dose groups, without a difference between the dosage groups (*p* 0.80). After 90 days, the T cell responses of both arms reduced but were still higher than the baseline, with a median of 194 SFU/10^6^ PBMCs (IQR 60–284) in the half-dose and 112 SFU/10^6^ PBMCs (56–228) in the full-dose groups.

## 4. Discussion

Booster with an mRNA vaccine, BNT162b2, after an AZD1222 primary series showed enhanced immunological outcomes for at least 3 months after the booster, as shown by the sVNT against the omicron and delta variants, anti-S-RBD IgG, and cell-mediated immune response using the IFNγ ELISpot assay. Non-inferiority responses were seen between the half-dose 15 µg of BNT162b2 and full-dose 30 µg arms. The immunogenicity responses between the half-dose vs. full-dose groups were not different among the older age group, but there was a higher response of anti-S-RBD IgG among adults aged from 18–59 years who received full-dose BNT162b2.

Since January 2022, omicron variants have been the main cause of the COVID-19 pandemic [2]. The vaccine effectiveness against symptomatic infection study in England [24] showed lesser protection against the omicron variant, with 48.9% effectiveness at 2–4 weeks after the second dose of AZD1222. The protective effect in AZD1222 recipients declined to nearly no protection against the omicron variant after 20–24 weeks [24]. Considering the reduced protection against the omicron variant, booster doses are needed. The U.S. Food and Drug Administration has shortened the interval for booster doses from 6 months [14] to 5 months [25] in BNT162b2 or mRNA-1273 primary-series recipients since January 2022. The Joint Committee on Vaccination and Immunisation (JCVI), United Kingdom, advised to shorten the interval from primary to booster vaccinations from 6 to 3 months [26]. With a shorter interval between primary vaccination and booster dose, there is a public health threat of vaccine supply shortage, especially among developing countries, e.g., in Africa continent with the coverage of primary vaccination being lower than 20% of the population [27].

BNT162b2 or mRNA-1273 booster in adults post AZD1222 vaccination showed improved protection against the omicron variant [24]. The increased neutralizing activity following booster vaccination might play a major role, since the neutralizing antibody levels were demonstrated to be predictive of vaccine efficacy [17]. Despite the lack of an omicron-specific vaccine, the mRNA vaccine booster offered protective benefits. Half-dose BNT162b2 boosters in AZD1222-primed individuals showed the ability to boost immune responses, both antibody and cellular-mediated, with anti-spike IgG GM of 16,045 ELU/mL and cellular response to wild type of 135.9 SFU/10^6^ PBMCs [11]. Our study showed that, after boosting with a full dose of BNT162b2, the geometric mean of neutralizing titer against the omicron variant was 67.3% inhibition.

A fractional low dose of BNT162b as a booster dose has been studied in Thailand among adults who received a primary series of the CoronaVac vaccine [28]. The study was conducted among 222 healthy adults with a median age of 39.1 years who received two-dose CoronaVac for 6 months. The neutralizing antibody against the omicron variant was 71.2% inhibition vs. 53.5% inhibition among participants who received 15 μg BNT162b2 vs. 30 μg BNT162b2, respectively. The finding was similar for participants who received 50 μg of mRNA-1273 vs. 100 μg of mRNA-1273, respectively (61.1%, and 76.7%).

Another study conducted among 100 healthy adults who received a primary series of AZD1222 and received 15 µg vs. 30 µg of BNT162b2 as boosters showed a slightly lower immunogenicity response among half-dose vs. full-dose groups [16]. The anti-RBD-IgG concentrations were 1961 and 2363 BAU/mL at 2 weeks post-booster and significantly declined to 314 and 431 BAU/mL at 16–20 weeks post-booster, respectively. For pVNT against the omicron variant, participants who received a half-dose had a lower titer than those receiving a full dose of BNT162b2; (at 2 weeks post-booster: 232 vs. 521; at 16–20 weeks post-booster: 14 vs. 116).

Our study found that BNT162b2 mRNA boosters after AZD1222 primary series increased cell-mediated immune responses. This finding was similar to the report from the COV-BOOST study and provided a benefit over AZD1222 as a third dose, which did not boost cellular responses after a homologous AZD1222 primary series [11]. SARS-CoV-2-specific T cell responses were shown to be essential for viral clearance and providing immunological memory, and might provide protection against severe infection and death [29]. A recent study showed that, compared with the wild type, T cell responses to the omicron variant were preserved in the majority of cases and enhanced by booster vaccination [30]. The T cell cytokine responses, used to demonstrate responses to SARS-CoV-2, included IFNγ, TNFα, IL-2, and/or granzyme B [31]. In this study, IFNγ was used as a marker of the T cell response.

The most frequently reported reactogenicity of booster BNT162b2 in participants post two-dose AZD1222 was pain at the injection site in 71% of recipients. This was markedly higher than the previous report [11], but comparable to that post-BNT162b2 primary series [13]. A similar trend was observed in systemic reactogenicities e.g., headache, fatigue, myalgia, and arthralgia [13]. Half-dose BNT162b2 recipients experienced less myalgia, fever, moderate-to-severe local pain, headache, fatigue, and arthralgia than standard-dose recipients, which might lead to more acceptability of a booster dose.

The strengths of this study were the double-blinded, randomized, controlled study design and several immunogenicity outcomes, including omicron-variant sVNT and cellular responses. The limitation for generalizability is that we did not include immunocompromised patients or participants older than 70 years old. Half-dose BNT162b2 or 0.15 mL might be considered burdensome for real-world implementation; however, using low-dead-space syringes, this volume of vaccine was manageable. After 3 months of boosters, the neutralizing antibodies waned, which might reduce vaccine effectiveness against any infection, but with the cell-mediated immune response, vaccine effectiveness against hospitalization and severe COVID-19 should remain. The immunogenicity results presented in this study included anti-S-RBD IgG, which was based on RBD from wild-type SARS-CoV-2 and could not represent the binding antibody level against the RBD of the omicron variant. However, sVNT against the omicron variant was conducted and could demonstrate a neutralizing antibody against the omicron variant. Further study on the vaccine effectiveness of half-dose BNT162b2 boosters in the era of the omicron variant is required.

To provide protection against the omicron variant, COVID-19 vaccines might need to be updated [32]. Several omicron-specific vaccines are being developed with promising results against the omicron variant in animal models [33,34,35,36], but one with comparable result to the original vaccine [37]. Phase 2 and phase 3 clinical trials of mRNA-1273.529 [38] and BNT162b2 OMI are underway [39]. The multivalent vaccine, based on different variants, or pan-sarbecovirus vaccines are a call to action to alleviate this pandemic [32]. The Coalition for Epidemic Preparedness Innovations (CEPI) has funded the development of broadly protected vaccines against variants and other betacoronaviruses, including severe acute respiratory syndrome coronavirus (SARS-CoV), Middle East respiratory syndrome coronavirus (MERS-CoV), and SARS-CoV-2, the causes of recent outbreaks [40].

## 5. Conclusions

Half-dose BNT162b2 mRNA vaccine booster after AZD1222 primary series provided non-inferior immunogenicity responses, as shown by the sVNT against the omicron variant and cell-mediated immune response. During the shortage of vaccine supply, a fractional low dose should be considered for a booster vaccination program.

## Figures and Tables

**Table 1:** Baseline characteristics of study participants.

Characteristics	Total (*n* = 100)	Half-Dose BNT162b2 Booster (15 µg/Dose) (*n* = 50)	Standard-Dose BNT162b2 Booster (30 µg/Dose) (*n* = 50)
Age (years), median (IQR)	59.3 (33.4–65.5)	59.3 (32.2–65.5)	57.9 (33.6–65.8)
- Group 18–59 years	33.4 (28.1–43.0)	32.3 (28.1–42.1)	33.6 (29.7–43.4)
- Group 60–69 years	65.5 (63.3–67.8)	65.5 (63.2–67.8)	65.8 (64.0–67.8)
Female, *n* (%)	61 (61)	31 (62)	30 (60)
BMI (kg/m^2^), median (IQR)	23.3 (21.8–26.1)	22.8 (21.1–25.3)	23.8 (22.3–27.5)
Interval between 2nd dose of AZD1222 to BNT162b2 (days), median (IQR)	98 (92–128)	98 (92–131)	98 (92–126)
sVNT to delta variant (% inhibition), GM (95% CI)	32.8 (27.5–39.1)	36.9 (29.9–45.6)	29.2 (22.0–38.9)
sVNT to wild type (% inhibition), GM (95% CI)	39.4 (33.5–46.5)	42.8 (34.8–52.6)	36.4 (28.1–47.2)
Anti-S-RBD IgG (BAU/mL), GM (95% CI)	199.1 (170.1–233.0)	193.7 (156.2–240.2)	204.6 (161.5–259.3)

BMI: Body mass index; GM: Geometric mean; S-RBD: Spike receptor-binding domain; sVNT: Surrogate virus neutralization test; Baseline characteristics between participants who received half-dose BNT162b2 and standard-dose BNT162b2 showed no statistically significant difference (*p* > 0.05).

**Table 2:** Results of sVNT for variants of concern and anti-S-RBD IgG at day 14 and day 90 among adults who received half-dose or standard-dose BNT162b2 booster.

Immunogenicity Outcomes	Half Dose GM (95% CI) (*n* = 50)	Standard Dose GM (95% CI) (*n* = 50)	GMR (95% CI)
sVNT to omicron variant (% inhibition)			
Day 14	74.4 (68.8–80.5)	67.3 (57.9–78.1)	1.11 (0.93–1.30)
Day 90	22.3 (14.9–33.4)	20.4 (13.1–32.0)	1.09 (0.60–1.98)
sVNT to delta variant (% inhibition)			
Day 14	100.0 (99.6–100.4)	100.0 (99.8–100.3)	1.00 (0.99–1.00)
Day 90	75.7 (67.6–84.7)	79.9 (69.7–91.7)	0.95 (0.79–1.13)
Anti-S-RBD IgG (BAU/mL)			
Day 14	2329.8 (2109.3–2573.4)	2574.7 (2262.5–2929.9)	0.90 (0.77–1.06)
Day 90	549.6 (479.1–630.5)	676.1 (573.8–796.7)	0.81 (0.66–1.00)

GM: Geometric mean; GMR: Geometric mean ratio; S-RBD: Spike receptor-binding domain; sVNT: Surrogate virus neutralization test. Day 90: 46 participants in each arm due to lost to follow-up and COVID-19 infection.

## Data Availability

The data presented in this study are available upon reasonable request to the corresponding author.

## Supplementary Materials

The following supporting information can be downloaded at: https://www.mdpi.com/article/10.3390/vaccines10060914/s1, Supplementary Table S1. COVID-19 in participants post-primary 2-doses of AZD1222 and after BNT162b2 boosters, during omicron predominance, Supplementary Table S2. Local and systemic reactogenicities within 7 days after BNT162b2 booster in adult post-2 doses of AZD1222, according to half-dose and standard-dose group.
