# Supplementary material for: A Randomized Clinical Trial of a Fractional Low Dose of BNT162b2 Booster in Adults Following AZD1222
Source: Vaccines (Basel). 2022 Jun 8;10(6):914. doi: 10.3390/vaccines10060914 (PMC9230769; doi:10.3390/vaccines10060914)
Supplement: Supplementary file 1 [file vaccines-10-00914-s001.zip › vaccines-1704679-supplementary.pdf]

A randomized clinical trial of fractional low dose of BNT162b2 booster dose in adult following AZD1222

Rapisa Nantanee [1,2]

Watsamon Jantarabenjakul [1,3]

Peera Jaru-Ampornpan [4]

Pimpayao Sodsai [5]

Orawan Himananto [6]

Jitthiwa Athipunjapong [1]

Jiratchaya Sophonphan [7]

Sira Nanthapisal [8]

Nattiya Hirankarn [9]

Thanyawee Puthanakit [1]

on behalf of the study team*

[1] Center of Excellence in Pediatric Infectious Diseases and Vaccines, Department of Pediatrics, Faculty of Medicine, Chulalongkorn University, Bangkok, Thailand

[2] Pediatric Allergy and Clinical Immunology Research Unit, Division of Allergy and Immunology, Department of Pediatrics, Faculty of Medicine, Chulalongkorn University, Bangkok, Thailand

[3] Thai Red Cross Emerging Infectious Diseases Clinical Center, King Chulalongkorn Memorial Hospital, Bangkok, Thailand

[4] Virology and Cell Technology Research Team, National Center for Genetic Engineering and Biotechnology (BIOTEC), Pathum Thani, Thailand

[5] Center of Excellence in Immunology and Immune-mediated Diseases, Department of Microbiology, Faculty of Medicine, Chulalongkorn University, Bangkok, Thailand

[6] Monoclonal Antibody Production and Application Research Team, National Center for Genetic Engineering and Biotechnology (BIOTEC), Pathum Thani, Thailand

[7] The HIV Netherlands Australia Thailand Research Collaboration (HIV-NAT), The Thai Red Cross AIDS Research Centre, Bangkok, Thailand

[8] Department of Pediatrics, Faculty of Medicine, Thammasat University, Pathum Thani, Thailand

[9] Department of Microbiology, Faculty of Medicine, Chulalongkorn University, Bangkok, Thailand

Corresponding author: Thanyawee Puthanakit, MD

Center of Excellence in Pediatric Infectious Diseases and Vaccines, Department of Pediatrics, Faculty of Medicine, Chulalongkorn University

1873, Rama IV, Pathumwan, Bangkok, 10330, Thailand.

Telephone: +662 2564930

E-mail: thanyawee.p@chula.ac.th

*Additional study team members are listed in the Acknowledgments.

2 Supplementary Tables

Thai Clinical Trials Registry (thaiclinicaltrials.org): TCTR20211027002

Supplementary Table S1. COVID-19 in participants post primary 2-doses of AZD1222 and after BNT162b2 boosters, during omicron predominance.

No.	Sex	Age (years)	COVID-19 symptoms	Treatment	BNT162b2 booster dose	Interval after booster (days)	sVNT-omicron (%inhibition) at day 14	Anti-S-RBD IgG (BAU/ml) at day 14	ELISpot (SFU/10^6 PBMC) at day 14
**P1**	F	31	Asymptomatic	Favipiravir	Half	58	52.7	2385	1228
**P2**	F	68	URI	Andrographis paniculata, ivermectin	Half	79	77.8	2159	412
**P3**	M	42	URI	Andrographis paniculata	Half	87	99.3	3960	-
**P4**	M	68	URI	Favipiravir	Standard	84	23.3	828	360
**P5**	M	64	URI	Favipiravir	Standard	89	66.6	1381	-
**P6**	F	67	Asymptomatic	none	Standard	89	89.4	2337	-

F: Female; M: Male; URI: Upper respiratory tract illness.

Supplementary Table S2. Local and systemic reactogenicities‡ within 7 days after BNT162b2 booster in adult post 2 doses of AZD1222, according to half dose and standard dose group.

Reactogenicities	Total (N=100) N (%)	Half dose (N=50) N (%)	Standard dose (N=50) N (%)	P-value†
**Local reactogenicities**				
**Pain**	**71 (71)**	**32 (64)**	**39 (78)**	**0.19**
• **Grade 1**	48 (48)	22 (44)	26 (52)	
• **Grade 2**	20 (20)	10 (20)	10 (20)	
• **Grade 3**	3 (3)	-	3 (6)	
**Swelling**	**2 (2)**	**1 (2)**	**1 (2)**	**0.99**
• **Grade 1**	1 (1)	-	1 (2)	
• **Grade 2**	1 (1)	1 (2)	-	
**Erythema**	-	-	**-**	-
**Systemic reactogenicities**				
**Fever**	**3 (3)**	**-**	**3 (6)**	**0.24**
• **Grade 1**	2 (2)	-	2 (4)	
• **Grade 2**	1 (1)	-	1 (2)	
**Headache**	**29 (29)**	**14 (28)**	**15 (30)**	**0.07**
• **Grade 1**	24 (24)	14 (28)	10 (20)	
• **Grade 2**	5 (5)	-	5 (10)	
**Fatigue**	**37 (37)**	**20 (40)**	**17 (34)**	**0.05**
• **Grade 1**	30 (30)	19 (38)	11 (22)	

• **Grade 2**	7 (7)	1 (2)	6 (12)	
**Myalgia**	**37 (37)**	**16 (32)**	**21 (42)**	**0.04***
• **Grade 1**	30 (30)	16 (32)	14 (28)	
• **Grade 2**	6 (6)	-	6 (12)	
• **Grade 3**	1 (1)	-	1 (2)	
**Arthralgia**	**16 (16)**	**4 (8)**	**12 (24)**	**0.06**
• **Grade 1**	12 (12)	4 (8)	8 (16)	
• **Grade 2**	3 (3)	-	3 (6)	
• **Grade 3**	1 (1)	-	1 (2)	
**Vomiting**	**1 (1)**	**1 (2)**	**-**	**0.32**
• **Grade 2**	1 (1)	1 (2)	-	
**Diarrhea**	**6 (6)**	**4 (8)**	**2 (4)**	**0.68**
• **Grade 1**	5 (5)	3 (6)	2 (4)	
• **Grade 2**	1 (1)	1 (2)	-	

[†]Chi-square test

[‡]Adverse events grading according to U.S. Department of Health and Human Services F, CBER. Guidance for Industry Toxicity Grading Scale for Healthy Adult and Adolescent Volunteers Enrolled in Preventive Vaccine Clinical Trials September 2007 [Available from: https://www.fda.gov/media/73679/download. Accessed date 30 November, 2021].

*$p < 0.05$
